# Association of hearing and vision impairment with cognitive impairment in nursing home residents in Switzerland

**DOI:** 10.1007/s10433-025-00880-y

**Published:** 2025-08-07

**Authors:** Ania Mikos, Nataliya Solmssen, Alexander Seifert, Nathalie Giroud, Florian Riese

**Affiliations:** 1 Healthy Longevity Center, University of Zurich, Stampfenbachstrasse 73, 8006 Zurich, Switzerland; 2 Department of Computational Linguistics, University of Zurich, Zurich, Switzerland; 3 School of Social Work, University of Applied Sciences and Arts Northwestern Switzerland, Olten, Switzerland; 4 University Competence Center “Language and Medicine”, University of Zurich, Zurich, Switzerland

**Keywords:** Cognitive impairment, Sensory impairment, Vision loss, Hearing loss, Nursing home, Long-term care

## Abstract

**Purpose:**

The objectives of this study were to ascertain the prevalence of vision and/or hearing impairment and investigate their association with cognitive impairment in nursing home residents in Switzerland.

**Methods:**

The sample comprised individuals aged 65–105 (mean = 84.0 and SD = 7.2) newly admitted for long-term care in one of 715 Swiss nursing homes from 2010 to 2019 (*N* = 132,880). Items from the Minimum Data Set of the Resident Assessment Instrument Swiss Version 2.0 assessed occurrence of hearing impairment (HI), vision impairment (VI), and dual sensory impairment (DSI, both HI and VI). We conducted logistic regression analyses to examine associations of each sensory impairment to cognitive impairment, controlling for medical and demographic factors.

**Results:**

Sensory impairment was present in 57.28% of residents (HI 19.16%, VI 15.13%, and DSI: 22.99%) and cognitive impairment in 57.29%. The likelihood of cognitive impairment was greatest for DSI (prevalence ratio (PR) 1.58; 95% confidence interval (CI) 1.56–1.60), followed by HI (PR 1.35; 95% CI 1.33–1.37) and VI (PR 1.18; 95% CI 1.16–1.20). The average marginal effect for DSI on cognitive impairment exceeded the sum of effects for HI and VI. Stratified models revealed that male sex, younger age, and higher medical comorbidity were associated with increased likelihood of cognitive impairment in DSI.

**Conclusion:**

Sensory impairments are prevalent among newly admitted nursing home residents. While HI and VI are independently associated with cognitive impairment, an interactive burden emerges when they co-occur as dual sensory impairment.

**Supplementary Information:**

The online version contains supplementary material available at 10.1007/s10433-025-00880-y.

## Introduction

Population aging worldwide leads to increasing prevalence of cognitive impairment and dementia-burdening affected individuals, their caregivers and healthcare systems in general. Like the prevalence of cognitive impairment and dementia, the prevalence of hearing impairment (HI) (GBD 2019 Hearing Loss Collaborators 2021) and vision impairment (VI) (GBD 2019 Blindness and Vision Impairment Collaborators; Vision Loss Expert Group of the Global Burden of Disease Study 2021) increases with age. Both HI (Yu et al. 2024) and VI (Nagarajan et al. 2022) have been linked to cognitive decline and have been recognized as potentially modifiable risk factors for dementia (Livingston et al. 2024). Indeed, as many as 7% of dementia cases may be attributable to HI and 2% to untreated VI on a population level (Livingston et al. 2024). Dual sensory impairment (DSI, characterized by simultaneous occurrence of HI and VI) has also recently been implicated in increased dementia risk (Yoshida et al. 2025). Growing attention has turned to how co-occurring sensory impairments may impact cognitive function more broadly, including milder, pre-dementia forms of decline.

The frequent co-occurrence of hearing loss with vision impairment (Chia et al. 2006; Besser et al. 2018) may present unique challenges for older individuals. The concept of multimorbidity suggests that the presence of multiple chronic conditions can interact in ways that amplify health outcomes beyond the sum of individual effects (e.g., Tinetti et al. 2011; Fried et al. 1999). Thus, DSI may represent not only an additive burden but also a potential accelerant of cognitive decline. Understanding this relationship is essential for identifying at-risk populations and informing interventions aimed at preserving cognitive function in older adults. To that end, it is also important to identify other risk factors for cognitive impairment among individuals with sensory impairment.

The interplay of sensory and cognitive impairment appears particularly consequential in the highly multimorbid population of nursing home residents. In Canadian long-term care facilities (LTCFs), prevalence of HI and VI was approximately 15% and 22%, respectively (Guthrie et al. 2022). This prevalence was remarkably stable over time from 2010 to 2018 (with only a slight decrease in VI and increase in HI) (Guthrie et al. 2022). DSI was present in approximately 25% of Canadian LTCF residents (Guthrie et al. 2022) and has been associated with higher rates of prevalent cognitive impairment in cross-sectional analyses (Davidson and Guthrie 2019; Mitoku et al. 2016). Longitudinal research also suggests an association between DSI and cognitive decline. In a prospective study of LTCF residents in Hong Kong, those with DSI experienced significantly greater cognitive decline over time compared to those with single or no sensory impairment (Kwan et al. 2022). In a sample of European nursing home residents, DSI was associated with greater cognitive decline only in residents who were not socially engaged (Yamada et al. 2016). Despite these findings, it remains unclear whether the effect of dual sensory impairment on cognitive impairment in nursing home residents exceeds the additive effect of each individual sensory impairment. Moreover, little is known about which factors (e.g., sex, age, and medical comorbidity) may amplify the risk of cognitive impairment in the presence of sensory loss.

The overall objectives of this study were twofold. First, we evaluated the prevalence of HI, VI, and DSI in newly admitted nursing home residents in Switzerland. Second, we explored the association of each sensory impairment with cognitive impairment. The aims of sensory-cognitive association analyses were to determine whether the effect of dual sensory impairment exceeds the additive effects of hearing and vision impairment and to characterize the moderating effects of sex, age group, and medical comorbidity count on these associations.

## Methods

### Study population and data source

We used data from the Resident Assessment Instrument-Minimum Data Set (RAI-MDS) for long-term care facilities, Swiss Version 2.0 (Anliker and Bartelt 2015). The RAI-MDS was originally developed for the US nursing home context (Morris et al. 1990), and versions of it are now widely used as assessment tools internationally in a variety of settings. The research database without personally identifying information was generated by BESA QSys AG, the company managing the RAI-MDS system in Switzerland. For this study, residents aged 65–105 with RAI-MDS admission assessment between the years of 2010 and 2019 (*N* = 134,670) were included. According to the RAI-MDS protocol, a significant correction of a prior full assessment is completed if a facility identifies a major error in a previously submitted comprehensive assessment. For the present dataset, the corrected assessment was used in cases where one correction assessment directly followed the admission assessment (*N* = 7592). Cases with multiple consecutive corrected assessments following the admission assessment were excluded (*N* = 545). Residents were excluded if they were comatose (*N* = 132) or had cerebral palsy (*N* = 523). Lastly, those residents with missing values in any of the variables in the full model (*N* = 590) were excluded. The final sample comprised a single admission assessment for each of 132,880 residents.

### Measurements

The presence of a HI was identified by a single item on the assessment that scores corrected hearing ability (i.e., with the use of hearing aids or other devices). The variable was scored on a 4-point scale from 0 (no impairment) to 3 (highly impaired). From the year 2016, the variable was changed to use a 5-point scale from 0 (adequate) to 4 (no hearing present). For both versions of the variable, a score of 1 (indicating minimal difficulty) or higher was used to indicate the presence of HI. RAI-MDS ratings of hearing were shown to significantly correlate with audiometrically measured pure-tone thresholds in a small study (Hopper et al. 2016).

VI was identified by a single item on the assessment that scores visual ability in adequate lighting with the use of their typical assistive device (e.g., glasses and magnifying glass) from 0 (no impairment) to 4 (severely impaired). The RAI-MDS rating of visual status was associated with both distance and near visual acuity and contrast sensitivity in a US sample (Swanson et al. 2009). A score of 1 or higher indicated VI, and this cohort included only those with a VI and not HI. DSI included individuals with both HI and VI, while the category of no sensory impairment (NSI) included individuals with scores of 0 for both HI and VI.

Cognitive function was measured using the cognitive performance scale (CPS) which is constructed from RAI-MDS items (Morris et al. 1994). It ranges from no cognitive impairment (level 0) to very severe impairment (level 6) and corresponds closely to Mini-Mental State Examination scores (Morris et al. 1994; Hartmaier et al. 1995). A cut point of two or greater has been validated as an indicator of cognitive impairment in nursing home samples and was, therefore, used in the present study to classify cognitive impairment (Hartmaier et al. 1995; Paquay et al. 2007). The RAI-MDS diagnostic categories “Alzheimer’s disease” and “Dementia other than Alzheimer’s disease” were used to determine the presence of a dementia diagnosis.

Performance on personal activities of daily living (ADL) tasks was rated with the ADL Hierarchy Scale derived from the RAI-MDS (Morris et al. 1999). The scale has seven levels ranging from total independence (level 0) to total dependence (level 6). For the purposes of this study, a score of 2 or higher (limited assistance for at least one ADL required) was used to define impairment.

The Depression Rating Scale (DRS) of the RAI-MDS screens for signs/symptoms of depression (Burrows et al. 2000). It is derived by creating a summary score across seven items and ranges from 0 to 14, with higher scores indicating more depressive symptoms. A DRS score of 3 or higher, as used in the current analysis is a valid measure of clinically meaningful symptoms of depression (Burrows et al. 2000; Martin et al. 2008).

Medical comorbidity was assessed based on the presence of six chronic conditions: hypertension, diabetes, coronary heart disease, stroke, emphysema/COPD, and cancer. The variable was dichotomized to indicate the presence of two or more conditions.

### Analysis

All analyses were performed with R version 4.5.1. Baseline characteristics were compared among the four sensory groups and analyzed using a one-way analysis of variance (ANOVA) for the continuous variable of age and a *χ*^2^ test for categorical variables. Next, logistic regression analysis was conducted to evaluate the association between sensory and cognitive impairment. We first calculated a crude model predicting cognitive impairment from the sensory status variable with four levels (NSI, HI, VI, and DSI) without any covariates. This was followed by a model that adjusted for relevant covariates including age group (65–74 years, 75–84 years, and ≥ 85 years), sex, medical comorbidity count (0–1 and ≥ 2), presence of depressive symptoms, ADL impairment, hearing aid use, and visual aid use.

Odds ratios may approximate the risk or prevalence ratio when the outcome is rare (e.g., ≤ 10%), but they overestimate these if the condition has a high or moderate incidence (e.g., > 10%) (Schmidt and Kohlmann 2008; Tamhane et al. 2017; Gnardellis et al. 2022). Overestimation may be further inflated with the inclusion of covariates and estimation of interaction effects (Gnardellis et al. 2022; Knol and VanderWeele 2012). Because our outcome variable, cognitive impairment, is relatively common in the nursing home setting, we provide prevalence ratios and average marginal effects (AMEs). These provide more intuitive measures of association and are less sensitive to model specifications than odds ratios (Knol and VanderWeele 2012; Norton et al. 2019; Norton and Dowd 2018). The AME is defined as the difference in the predicted probability of the outcome (i.e., cognitive impairment) for a discrete change from 0 to 1 of a binary explanatory variable (e.g., going from NSI to HI) (Norton et al. 2019; Norton and Dowd 2018). For the fully adjusted model, AMEs were calculated by evaluating the model at values of the binary covariates that reflect their proportions in the sample and the categorical age covariate representing the distribution of individuals across the defined age groups in the data. This ensures that the predictions represent the actual sample being studied. Thus, AMEs quantify the adjusted change in predicted probability of cognitive impairment associated with a given sensory impairment status relative to no sensory impairment.

For each model, we calculated a concordance statistic (C-statistic) to assess the goodness of fit of the model. We considered that a C-statistic equal to or < 0.5 was indicative of a poor model, a value between 0.7 and 0.8 was indicative of a good model, a value between 0.8 and 0.9 was indicative of a strong model, and a value over 0.9 was indicative of a highly robust model (LaValley 2008).

Next, we explored whether the association between sensory and cognitive impairment differed by sex, age group, or medical comorbidity count (0–1 and ≥ 2). Logistic regression models were run with two-way multiplicative interaction terms (e.g., sensory status X sex) to assess for a significant difference (*p* < 0.05) in the − 2LL values of the models with and without the interaction terms. AMEs were estimated to assess changes in the probability of cognitive impairment associated with sensory status and were compared across subgroups (e.g., male vs female) to evaluate potential effect modification.

## Results

### Participant characteristics

Of the 132,880 participants included in the analysis, 42.7% were classified as having no sensory impairment (NSI), while 57.3% had hearing and/or visual impairments. Of these, 19.2% had only HI, 15.1% only VI, and 23.0% had DSI.

A one-way ANOVA revealed a significant difference in age (continuous variable) by sensory status [*F*(3, 132,876) = 3524, *p* < 0.001, *SS* = 507,126, MS = 169,042]. Post hoc analysis, using Tukey’s HSD test to adjust for multiple comparisons, revealed significant differences in mean age between all sensory status groups (i.e., NSI < VI < HI < DSI, *p* < 0.01 for all comparisons). This suggests an association between average age and sensory impairments, with those having dual sensory impairments being the oldest on average.

A series of Chi-squared tests revealed significant associations between sensory status and categorical demographic and medical variables (see Table 1 for full results). Post hoc analyses of the standardized residuals for each cell were performed to identify whether observed frequencies were higher or lower than would be expected by chance. HI only was significantly associated with being 85 or older, male, having a dementia diagnosis, cognitive impairment, ADL impairment, and the use of both hearing and visual aids. VI only was more highly represented in the younger age groups (65–74 and 75–84) and was significantly associated with being female, having ADL impairment, depressive symptoms, and use of visual aids. VI was less commonly associated with cognitive impairment and with hearing aid use. Finally, DSI was associated with being 85 or older, male, having a dementia diagnosis or cognitive impairment, depressive symptoms, ADL impairment, and using hearing aids.

**Table 1:** Demographic characteristics by sensory impairment

	Total	NSI (42.7%)	HI (19.2%)	VI (15.1%)	DSI (23.0%)	*p*-value
Age, years (SD)	84.0 (7.2)	82.1 (7.1)	85.9 (6.5)	83.0 (7.1)	86.5 (6.8)	< 0.001
Age group						< 0.001
65–74 (%)	11.6	16.4^a^	6.0^b^	13.8^a^	5.8^b^	
75–84 (%)	36.6	42.5^a^	30.8^b^	40.1^a^	28.1^b^	
≥ 85 (%)	51.8	41.1^b^	63.2^a^	46.1^b^	66.1^a^	
Female (%)	65.7	67.9^a^	61.0^b^	69.1^a^	63.3^b^	< 0.001
Medical comorbidity (≥ 2; %)^c^	35.1	35.1	35.4	35.6	34.4	0.023
Dementia (%)	41.0	35.9^b^	42.3^a^	40.7	49.5^a^	< 0.001
Cognitive impairment (%)	57.3	45.1^b^	62.0^a^	56.3^b^	76.8^a^	< 0.001
Depressive symptoms (%)	21.7	17.7^b^	21.5	23.2^a^	28.4^a^	< 0.001
ADL self-hierarchy scale (≥ 2; %)	74.7	67.3^b^	75.5^a^	76.6^a^	86.3^a^	< 0.001
Use hearing aid (%)	15.2	7.5^b^	29.9^a^	4.8^b^	24.1^a^	< 0.001
Use visual aid (%)	69.4	67.8^b^	70.8^a^	74.1^a^	67.9^b^	< 0.001

^a^Statistically significant association with FDR correction (residual > 1.96)

^b^Statistically significant association with FDR correction (residual < 1.96)

^c^Includes hypertension, diabetes, coronary heart disease, stroke, emphysema/COPD, and cancer

### Association of sensory impairments with cognitive impairment

Of the 132,880 nursing home residents assessed at admission, 57.3% were cognitively impaired. Logistic regression revealed that VI and HI were significantly associated with cognitive impairment (Table 2). The point estimate for the prevalence ratio was highest among residents with coexisting hearing and vision impairment (DSI). Even after adjustment for potential confounders, residents with DSI demonstrated a greater likelihood of cognitive impairment (PR 1.58; 95% CI 1.56–1.60) than those with only HI (PR 1.35; 95% CI 1.33–1.37) or only VI (PR 1.18; 95% CI 1.16–1.20).

**Table 2:** Unadjusted and adjusted associations between sensory impairment and cognitive impairment

	Unadjusted		Adjusted	
	PR (95% CI)	AME (95% CI)	PR (95% CI)	AME (95% CI)
*NSI (reference)*				
HI	1.38 (1.36–1.39)	0.17 (0.16–0.18)	1.35 (1.33—1.37)	0.16 (.16-.17)
VI	1.25 (1.23–1.27)	0.11 (0.10–0.12)	1.18 (1.16–1.20)	0.08 (.08-.09)
DSI	1.70 (1.68–1.72)	0.32 (0.31–0.32)	1.58 (1.56–1.60)	0.27 (.27-.28)
Sex (reference: male)			0.93 (0.92–0.94)	-.04 (-.05- -.04)
Age 75–84 (reference: 65–74)			1.07 (1.05–1.08)	0.04 (0.03—0.05)
Age ≥ 85 (reference: 65–74)			1.04 (1.02–1.05)	0.02 (0.01—0.03)
Medical comorbidity (≥ 2)			0.90 (0.89—0.91)	-.06 (-.06—-.05)
Depressive symptoms			1.31 (1.30–1.33)	0.17 (0.16—0.18)
ADL impairment			1.58 (1.56–1.60)	0.23 (0.23—0.24)
Hearing aid use			0.84 (0.83—0.86)	-.09 (-.10—-.08)
Visual aid use			0.84 (0.84—0.85)	-.10 (-.11—-.09)
C-statistic	0.64		0.73	

*PR* prevalence ratio, *AME* average marginal effect, *CI* confidence interval

To assess the potential for residual confounding related to differential sensory aid use, we compared aid use by cognitive status. Hearing aid use was reported by 14.1% of residents with cognitive impairment and 16.6% of those without. Vision aid use was reported by 64.6% of residents with cognitive impairment and 75.8% of those without. These differences were statistically significant for both hearing aid use (*χ*^2^(1) = 160.3, *p* < 0.001) and vision aid use (*χ*^2^(1) = 1922.5, *p* < 0.001), suggesting that residents with cognitive impairment were less likely to use sensory aids.

Average marginal effects (AMEs) quantify the average change in the predicted outcome associated with a change from NSI to either HI, VI, or DSI. In the adjusted model, a change from NSI to HI was associated with a 16% increase in the likelihood of cognitive impairment while a change from NSI to VI was associated with an 8% increase. The AME of HI was significantly greater than the AME of VI (*z* = 15.01, two-tailed *p* < 0.001).

**Fig. 1:**
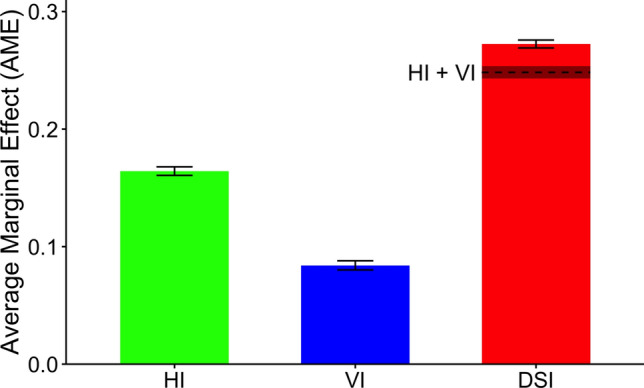
Average marginal effects on cognitive impairment for HI, VI, and DSI relative to NSI. Error bars represent standard errors. Exact values: HI = 0.16 (SE = 0.004), VI = 0.08 (SE = 0.004), and DSI = 0.27 (SE = 0.003). The dashed line represents the simple additive effect of HI and VI. The amount by which the AME for DSI exceeds the dashed line represents the supra-additive effect [AME_DSI_—(AME_HI_ + AME_VI_) = 0.024]

To further explore the combined association of hearing and visual impairments, we tested for additive interaction on the probability scale—assessing whether the joint association of HI and VI (i.e., DSI) with cognitive impairment exceeded the sum of their individual associations. The AME for DSI significantly exceeded the sum of AMEs for HI and VI (AME_DSI_—(AME_HI_ + AME_VI_) = 0.024, *SE* = 0.006, *z* = 3.82, *p* < 0.001), indicating a supra-additive effect of DSI on cognitive impairment (see Table 2 and Fig. 1).

### Effect modification analysis

**Fig. 2:**
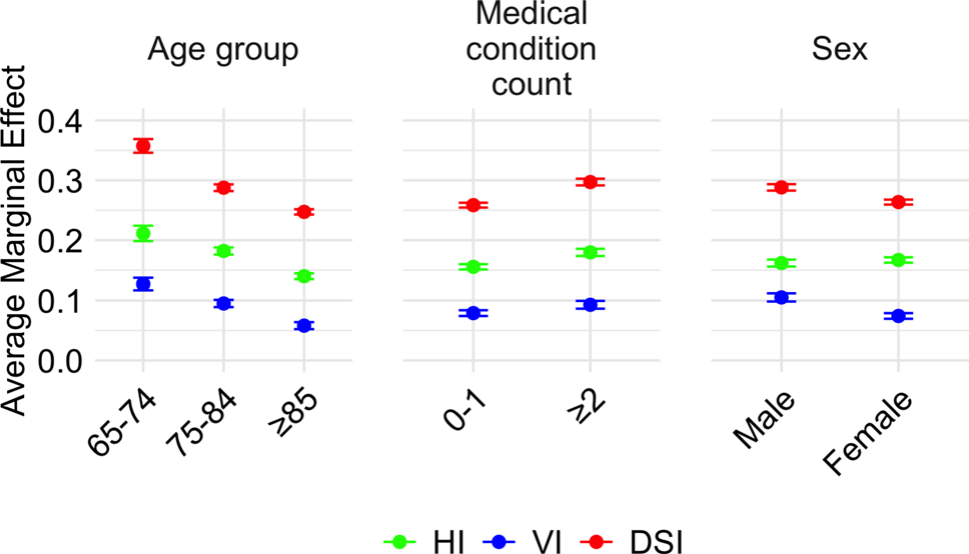
Average marginal effects of sensory impairments on cognitive impairment by subgroups for each of three interaction models. Error bars represent standard errors

To assess potential effect modification by sex, age, or medical comorbidity, three logistic regression models were constructed in which each of these variables interacted with sensory status in predicting cognitive impairment. All other covariates were retained from the fully adjusted model. Likelihood ratio tests indicated that models including interaction terms provided a significantly better fit than the fully adjusted model with no interaction term: sex (*χ*^2^(3) = 41.64, *p* < 0.001), age group (*χ*^2^(6) = 122.12, *p* < 0.001), and medical comorbidity count (*χ*^2^(3) = 17.36, *p* < 0.001). Since interaction models significantly improved model fit, we further examined these interactions using AMEs stratified by each moderating variable (Fig. 2 and Supplementary Table 1).

To decompose the interaction effects, we conducted statistical tests of subgroup differences in AME values (Supplementary Table 1). The effect of sensory impairment on cognitive impairment differed between males and females such that among those with DSI or VI, males had a significantly greater likelihood of cognitive impairment than did females. Among those with DSI or with HI, those with ≥ 2 medical conditions had a higher likelihood of cognitive impairment than those with 0–1 medical conditions. Analysis of the age interaction showed that for each sensory group, the older age group had significantly less likelihood of cognitive impairment than the next younger age group (e.g., ≥ 85 vs. 75–84 and 75–84 vs. 65–74).

### Sensitivity analysis

We conducted a sensitivity analysis using a higher CPS threshold (≥ 3), which corresponds to at least moderate cognitive impairment. This approach aimed to reduce potential misclassification and increase specificity based on prior concerns about potential inaccuracy of CPS rating raised in Swiss acute care settings (Bula and Wietlisbach 2009). Results of the adjusted logistic regression model were largely comparable to those of the original analysis. With a CPS cut-off of ≥ 3, 39.9% of the sample exhibited cognitive impairment. Average marginal effects and prevalence ratios for sensory status remained similar in magnitude and direction to the original model (Supplementary Table 2). The magnitude of the supra-additive effect of DSI on cognitive impairment increased slightly compared to the original analysis (AME_DSI_—(AME_HI_ + AME_VI_) = 0.039, SE = 0.006, *z* = 6.31, *p* < 0.001).

## Discussion

We investigated the prevalence of HI, VI, and DSI and their association with cognitive impairment in a large dataset of N = 132,880 newly admitted nursing home residents in Switzerland. Our main findings are a comparable prevalence of sensory impairment to previous reports from LTCFs in other countries and a supra-additive effect of DSI on cognitive impairment exceeding the independent effects of HI and VI. Finally, DSI was more strongly associated with cognitive impairment in younger age groups, males, and those with more medical diagnoses.

### Prevalence of sensory impairment in nursing homes

Any type of visual and/or hearing impairment was present in 57.3% of our sample (HI 19.2%, VI 15.1%, and DSI: 23.0%), i.e., more than half of nursing home residents were rated by trained nurses as having relevant impairments despite the use of hearing or vision aids—underlining the public health importance of sensory impairment. Our prevalence findings are comparable to those from Canadian nursing homes, where > 1.5 million RAI LTCF assessments were analyzed in the largest similar study so far (prevalence approximately HI 15%, VI 22%, and DSI 25%) (Guthrie et al. 2022). An analysis of RAI data from 59 nursing homes indicated wide variation of sensory impairment prevalence across eight European countries (not including Switzerland) (Yamada et al. 2014). Compared to their overall findings (HI 12.3%, VI 19.5%, and DSI: 31.8%), we found a substantially lower prevalence of DSI (Yamada et al. 2014). Similarly, RAI LTCF data from four countries (Canada, the US, Finland, and Belgium) identified a large variation between countries and a prevalence of DSI ranging from 9.7% in the US to 33.9% in Belgium (Guthrie et al. 2016). Such variation between countries is striking and may either be due to different national assessment practices or represent real differences in prevalence. Future research employing validation of RAI-MDS sensory assessments could help address this question.

### Single sensory impairments and cognitive impairment

We found an association of both HI and VI with cognitive impairment in our population—consistent with the previous literature (Yu et al. 2024; Nagarajan et al. 2022). Our study thus provides further evidence for the public health importance of sensory impairment both directly due to its high prevalence and indirectly through its association with cognitive impairment.

In our analyses, hearing aid use rates were well below 100%, yet the use of both hearing and visual aids was associated with a lower probability of cognitive impairment. Similarly, a cross-sectional study of a nationally representative sample of older adults in the US found that moderate to severe hearing loss was linked to a higher prevalence of dementia, whereas hearing aid use was associated with lower dementia prevalence (Huang et al. 2023). While cross-sectional associations could simply reflect a tendency for healthier individuals to use sensory aids, other lines of evidence support their potential to mitigate the risk of cognitive decline. Findings from an observational longitudinal study of long-term care facility RAI data in Hong Kong suggested that visual aids—but not hearing aids—were associated with a reduced risk of cognitive decline (Kwan et al. 2022). The randomized controlled ACHIEVE trial tested audiological counseling and provision of hearing aids against a control intervention of health education. Although the trial did not meet its primary endpoint of reducing cognitive decline, a significant reduction in 3 years cognitive decline was demonstrated among a pre-specified high-risk subgroup (Lin et al. 2023). Likewise, in the randomized controlled ENHANCE trial, hearing aid users exhibited significantly better cognitive performance 3 years after fitting, suggesting that hearing intervention may help delay cognitive decline in older adults (Sarant et al. 2023).

Given the direct benefits of sensory aids on areas such as communication and social functioning as well as their potential role in mitigating cognitive decline as indicated by randomized prospective trials, we strongly advocate for their promotion. In the nursing home context, this would require providing the necessary assistance for their use, ideally through close on-site collaboration with specialists in low vision (Seifert and Nosch 2024) and hearing services. Future research should explore whether such approaches are feasible and effective.

### Dual sensory impairment and cognition

We identified a particularly strong association of DSI with cognitive impairment—in line with previous findings (Davidson and Guthrie 2019; Mitoku et al. 2016; Kwan et al. 2022; Yamada et al. 2016; Fuller-Thomson et al. 2022). Furthermore, the estimated marginal effect exceeded the added effect estimates of the single impairments, suggesting a supra-additive association with cognitive impairment. DSI was associated with a 2.4% higher likelihood of cognitive impairment than would be expected from the sum of the associations for HI and VI alone. In relative terms, this corresponds to an approximately 10% increase in the likelihood of cognitive impairment with DSI compared to the (hypothetical scenario of) merely adding the effects of HI and VI.

Several theoretical frameworks have been proposed to explain the robust associations observed between sensory and cognitive impairments in older adults. The common cause hypothesis suggests that age-related declines across sensory and cognitive domains may reflect shared neurobiological or vascular processes (Lindenberger and Baltes 1994). In that vein, hearing and visual impairments may serve as markers of biological aging or of general health, reflecting underlying systemic processes that contribute to both sensory and cognitive decline (Chia et al. 2006; Fischer et al. 2016). The sensory deprivation hypothesis posits that chronic sensory loss may lead to long-term structural and functional brain changes that contribute to cognitive decline, while the information degradation hypothesis suggests that diminished sensory input increases cognitive effort during perception, potentially diverting resources away from higher-order functions (for review, see Whitson et al. 2018).

The late-life sensory compensation hypothesis offers an additional explanatory framework, particularly relevant in the context of dual sensory impairment (Wettstein et al. 2018). This hypothesis proposes that when only one sensory modality is impaired, individuals can compensate by relying on the other; however, with DSI, this compensation is no longer possible, leading to disproportionately worse cognitive outcomes. In a population-based sample of healthier, community-dwelling adults, hearing and vision impairments were associated with additive—but not supra-additive—effects on cognition (Phillips et al. 2022), perhaps reflecting preserved compensatory capacity. In contrast, our nursing home sample, which experiences more severe impairment and higher morbidity, showed a supra-additive association between DSI and cognitive impairment. This may signal the breakdown of compensatory capacity in the context of broader health decline.

In real-world terms, even apparently modest differences like the 2.4% absolute increase or 10% relative increase due to DSI could have substantial public health implications, particularly in populations where sensory impairments and cognitive decline are highly prevalent such as nursing home residents. In line with previous findings (Fuller-Thomson et al. 2022), the effect of sensory impairments seemed to be stronger in the younger age groups, possibly making younger nursing home residents a particularly promising target group for interventions. Future research should address if early-onset DSI sets affected individuals on a particularly steep trajectory of cognitive decline.

### Strengths and limitations

Limitations of our study include the cross-sectional nature of the analysis and the geographic restriction to Switzerland. In addition, the assessment of hearing, vision, and cognition relied on the RAI-MDS 2.0 instrument, a clinical assessment tool with known limitations. Sensory status is evaluated through observation and judgment by trained staff, which may introduce variability in assessment practices. Moreover, the presence of cognitive impairment may hinder the accurate assessment of hearing and vision—due to communication difficulties, reduced engagement, or behavioral symptoms—potentially leading to overestimation of sensory impairment in cognitively impaired residents. Conversely, sensory impairments may interfere with the assessment of cognitive function and artificially inflate CPS scores. These bidirectional sources of measurement bias may affect the observed associations. In addition, although the models adjusted for hearing and vision aid use, the lower use of sensory aids among cognitively impaired residents raises the possibility of residual confounding.

Although the Cognitive Performance Scale (CPS) is widely used and validated in nursing home populations, it is based on staff observation rather than direct neuropsychological testing, which may limit sensitivity to early or subtle cognitive changes. Similarly, our measure of medical comorbidity was based on a limited set of six diagnoses and may underestimate the true burden of multimorbidity, particularly if some conditions were underreported. Together, these limitations highlight potential sensitivity issues and underscore the need for further research employing validated, objective measures of sensory, cognitive, and general health status.

Our study has several strengths, including the use of a large routine care dataset that builds on the internationally used RAI system. Analytically, we used prevalence ratios (PRs) instead of the frequently reported odds ratios (ORs). For high prevalence conditions (such as cognitive impairment in nursing homes), ORs tend to overestimate relative risk (Schmidt and Kohlmann 2008; Tamhane et al. 2017; Gnardellis et al. 2022). While both ORs and PRs allow for covariate adjustment, PRs (and average marginal effects, AMEs) provide a more direct and interpretable measure of risk or probability because they incorporate the actual distribution of covariates in the population. Thus, our conclusions regarding the likelihood of cognitive impairment related to sensory impairment are based on a more interpretable and population-relevant measure of risk, enhancing their validity and applicability. Another strength of our study is the use of AMEs to disentangle the additive and supra-additive effect conveyed by DSI over single impairments—and to our knowledge is the first to demonstrate a supra-additive effect.

## Conclusions

Our study highlights the important public health challenge posed by dual sensory impairment, a condition affecting more than one in five nursing home residents in our sample. We found that having both hearing and visual impairments is associated with a greater likelihood of cognitive impairment than either sensory impairment alone. Considering the high prevalence and impact of sensory impairments, interventions targeting even a single sensory modality may play a valuable role in the comprehensive care of this population.

## Supplementary Information

Below is the link to the electronic supplementary material.

Supplementary file 1 (DOCX 15 KB)

## Data Availability

Due to contractual obligations with BESA QSys AG the original data used in this study cannot be shared publicly.
