# Supplementary material for: Association of hearing and vision impairment with cognitive impairment in nursing home residents in Switzerland
Source: Eur J Ageing. 2025 Aug 7;22(1):39. doi: 10.1007/s10433-025-00880-y (PMC12332150; doi:10.1007/s10433-025-00880-y)
Supplement: Supplementary file 1 — (DOCX 15 KB) [file 10433_2025_880_MOESM1_ESM.docx]

**Supplementary Table 1**. Average Marginal Effects (AMEs) of sensory status on cognitive impairment, stratified by each of three moderating variables. The “Difference” column represents the difference in AMEs between subgroups. The z-value and p-value indicate whether each difference is statistically significant. P-values for difference values in each interaction model were adjusted using the **Holm method** to control for family-wise error rate.

Panel A: Effect modification by age group

|  |  |  |  | 75–84 vs 65–74 | | | ≥85 vs 75–84 | | |
| --- | --- | --- | --- | --- | --- | --- | --- | --- | --- |
|  | 65–74 | 75–84 | ≥85 | Difference (SE): | z-value | *p*-value | Difference (SE) | z-value | *p*-value |
| DSI | 0.36 (0.01) | 0.29 (0.01) | 0.25 (0.00) | -0.07 (0.01) | -5.49 | <0.01 | -0.03 (0.01) | -3.97 | <0.01 |
| HI | 0.21 (0.01) | 0.18 (0.01) | 0.14 (0.00) | -0.03 (0.01) | -2.07 | 0.04 | -0.03 (0.01) | -4.04 | <0.01 |
| VI | 0.13 (0.01) | 0.10 (0.01) | 0.06 (0.01) | -0.03 (0.01) | -2.71 | 0.01 | -0.03 (0.01) | -3.43 | <0.01 |

Panel B: Effect modification by sex

|  | Male | Female | Difference (SE) | z-value | *p*-value |
| --- | --- | --- | --- | --- | --- |
| DSI | 0.29 (0.01) | 0.26 (<0.01) | 0.02 (0.01) | 3.62 | <0.01 |
| HI | 0.16 (0.01) | 0.17 (<0.01) | -0.01 (0.01) | -0.69 | 0.49 |
| VI | 0.11 (0.01) | 0.07 (<0.01) | 0.03 (0.01) | 3.72 | <0.01 |

Panel C: Effect modification by medical comorbidity count

|  | 0-1 | ≥2 | Difference (SE) | z-value | *p*-value |
| --- | --- | --- | --- | --- | --- |
| DSI | 0.26 (<0.01) | 0.30 (0.01) | -0.04 (0.01) | -5.60 | <0.01 |
| HI | 0.16 (<0.01) | 0.18 (0.01) | -0.02 (0.01) | -3.23 | <0.01 |
| VI | 0.08 (<0.01) | 0.09 (0.01) | -0.01 (0.01) | -1.72 | 0.09 |

**Supplementary Table 2.** Unadjusted and adjusted associations between sensory impairment and cognitive impairment (CPS ≥ 3) in sensitivity analysis

|  | Unadjusted | | Adjusted | |
| --- | --- | --- | --- | --- |
|  | PR (95% CI) | AME (95% CI) | PR (95% CI) | AME (95% CI) |
| NSI (reference) |  |  |  |  |
| HI | 1.35 (1.32-1.37) | 0.10 (0.10-0.11) | 1.33 (1.31 - 1.36) | 0.10 (.10-.11) |
| VI | 1.34 (1.31-1.37) | 0.10 (0.09-0.11) | 1.25 (1.23-1.28) | 0.08 (.07-.09) |
| DSI | 1.90 (1.87-1.93) | 0.27 (0.26-0.28) | 1.71 (1.68-1.74) | 0.22 (.22-.23) |
| Sex (reference:  male) |  |  | 0.90 (0.89-0.92) | -.04 (-.05- -.04) |
| Age 75-84  (reference: 65-74) |  |  | 1.07 (1.04-1.09) | 0.03 (0.02 - 0.03) |
| Age ≥85 (reference:  65-74) |  |  | 0.99 (0.97-1.01) | 0.00 (-.01 - 0.00) |
| Medical  comorbidity (≥2) |  |  | 0.84 (0.83 - 0.86) | -0.07 (-.07 - -.06) |
| Depressive  symptoms |  |  | 1.40 (1.38-1.42) | 0.15 (.14 - .15) |
| ADL impairment |  |  | 2.20 (2.15-2.25) | 0.25 (.24-.26) |
| Hearing aid use |  |  | 0.79 (0.77 - 0.81) | -.09 (-.09 - -.08) |
| Visual aid use |  |  | 0.75 (0.74 - 0.76) | -.12 (-.13 - -.12) |
| C-statistic | 0.61 | | 0.72 | |

PR = Prevalence Ratio; AME = Average Marginal Effect; and CI = Confidence Interval
